# Pinpointing beta adrenergic receptor in ageing pathophysiology: victim or executioner? Evidence from crime scenes

**DOI:** 10.1186/1742-4933-10-10

**Published:** 2013-03-15

**Authors:** Gaetano Santulli, Guido Iaccarino

**Affiliations:** 1 Departments of Translational Medical Sciences and Advanced Biomedical Sciences, “Federico II” University, Naples, Italy; 2 Columbia University in the City of New York, Manhattan, , New York, NY, USA; 3 Multimedica Research Hospital, Milan, Italy; 4 Department of Medicine and Surgery, University of Salerno, Salerno, Italy

## Abstract

G protein-coupled receptors (GPCRs) play a key role in cellular communication, allowing human cells to sense external cues or to talk each other through hormones or neurotransmitters. Research in this field has been recently awarded with the Nobel Prize in chemistry to Robert J. Lefkowitz and Brian K. Kobilka, for their pioneering work on beta adrenergic receptors (βARs), a prototype GPCR. Such receptors, and β_2_AR in particular, which is extensively distributed throughout the body, are involved in a number of pathophysiological processes. Moreover, a large amount of studies has demonstrated their participation in ageing process. Reciprocally, age-related changes in regulation of receptor responses have been observed in numerous tissues and include modifications of βAR responses. Impaired sympathetic nervous system function has been indeed evoked as at least a partial explanation for several modifications that occur with ageing. This article represents an updated presentation of the current knowledge in the field, summarizing in a systematic way the major findings of research on ageing in several organs and tissues (crime scenes) expressing βARs: heart, vessels, skeletal muscle, respiratory system, brain, immune system, pancreatic islets, liver, kidney and bone.

## Introduction

The β adrenergic receptors (or adrenoceptors, ARs) belong to the guanine nucleotide-binding G protein-coupled receptor (GPCR) superfamily [1]. GPCRs with seven transmembrane helices are indisputably the most important drug targets in medicine and their molecular and structural characterization has recently been honored with the 2012 Nobel Prize for chemistry to Bob Lefkowitz and Brian Kobilka [2-7].

**Figure 1:**
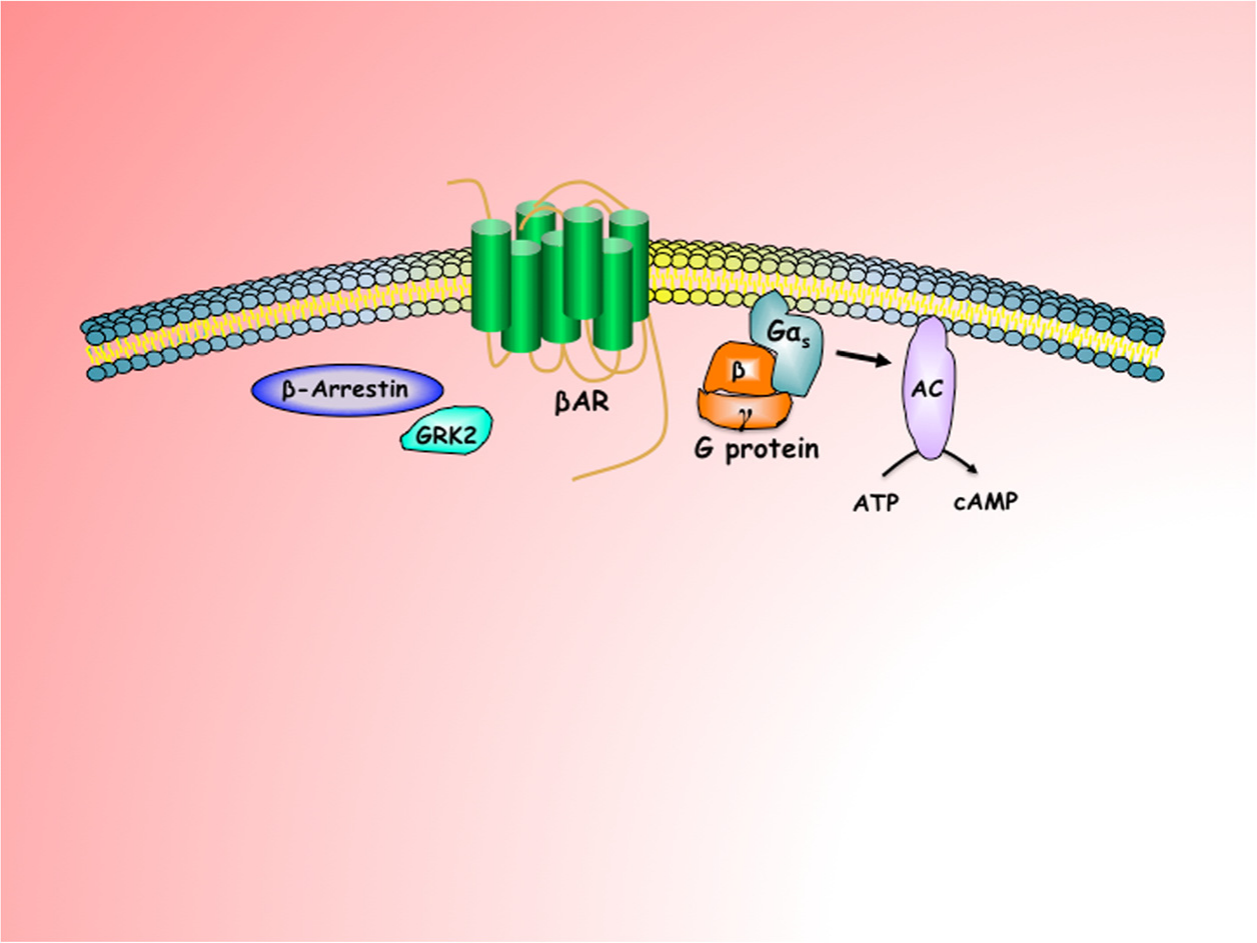
**Classical pathway of β**_**2**_**adrenergic receptor (β**_**2**_**AR) activating Gα**_**s**_**protein, which in turn activates the conversion of ATP in cAMP by the adenylate cyclase (AC).** An alternative signaling pathway involves Gα_i_ protein. G-protein-linked receptor kinase 2 (GRK2) and β-Arrestin participate in the desensitization process of the receptor.

βARs mediate physiological responses to catecholamines. There are three receptor subtypes in βAR family: β_1_AR is found at its highest levels in the heart, β_2_AR is distributed extensively throughout the body [8] and β_3_AR is mainly expressed in the white and brown adipose tissue [9,10]. These receptors consist of seven membrane-spanning domains, three intra- and three extracellular loops, one extracellular ammino-terminal domain, and one intracellular carboxy-terminal tail [11,12]. Ligand binding to the receptor promotes the exchange of bound guanosine diphoshate (GDP) for guanosine triphosphate (GTP) on the Gα subunit and subsequent dissociation of Gα from Gβγ, leading to the activation of Gα and release of Gβγ heterodimers. Thus, both Gα and Gβγ function as signaling mediators to directly interact with a variety of downstream effector proteins. There are several isoforms of each subunit of the trimeric G protein. In particular, β_2_AR is coupled to the Gα_s_ protein, which associates with the third intracellular loop of the βAR, resulting in activation of adenylyl cyclase, which in turn catalyzes the conversion of adenosine triphosphate to cyclic AMP, as depicted in Figure 1. All three βARs couple primarily to Gα_s_, but under certain conditions can also couple to Gα_i_[13]. Focusing on the β_2_AR, although the majority of its signaling occurs via Gα_s_ and subsequent cAMP-dependent mechanisms, there is evidence of other signaling schemes. The main alternative signaling pathway is the Gα_i_-dependent pathway, which ultimately results in the activation of the mitogen-activated protein kinase (MAPK) pathway [14-17]. Recent studies suggested that β_2_AR signaling might also occur via a G protein independent mechanism [18-21]. These paradigms of signaling may be observed in the same cell type occurring differently and according to the functional state of the cell. Therefore numerous conditions including chronic stimulation, acidosis, cell hypoxia and ageing can all modify the response to GPCR stimulus.

### Understanding ageing

Ageing is a complex process characterized by a gradual decline in organ functional reserves, which eventually reduces the ability to maintain homeostasis [22,23]. Several theories of ageing have been proposed and all of them relate to individual maintenance mechanisms. The somatic mutation theory relates to the failure of DNA repair; the free radical theory relates to the failure of defenses against reactive oxygen species; the autoimmune theory proposes that the immune system eventually fails to distinguish self from non-self antigens [24]; other researchers relate to the deleterious effects of toxic chemicals or to the loss of epigenetic controls, including DNA methylation [22,25,26].

In summary, the lifespan of each species seems to depend on the efficacy of maintenance of several biological processes and there is much evidence that such maintenance is more effective in long-lived, such as human, than in short-lived species [22,27]. The mechanisms underlying ageing have to be definitely looked at the cellular and molecular level, using a broad biological approach. Although the process seems to be irreversible and continuous, ageing itself does not mean pathology [25]. Indeed, ageing is a totally natural phenomenon and cannot be considered a pathological condition. However age-linked modifications indubitably pave the way for disease.

In this review we present an updated exposition of the current knowledge in the relationship between ageing and βARs, summarizing the major findings of research on ageing in several organs expressing βARs (Table 1). The senescence process is different in each tissue: the brain suffers from neurofibrillary degeneration and senile plaques; the vessels become rigid due to protein glycation and develop atheroma; renal function declines in parallel with the fall in the glomerular filtration rate due to a gradual decrease in the nephron pool; immune defenses become less effective due to the functional degradation of the lymphocytes and thymus involution. This article represents the first systematic report of the potential reciprocal regulation of ageing and βARs in various districts, providing both molecular and clinical implication for the use of common pharmaceuticals such as the βAR agents, both agonists and blockers, in elderly [28]. In particular, it is widely recognized that the incidence of adverse reactions to β-blockers is greater in hospitalized senescent patients than younger patients [29]. Of course, concomitant disorders, polypharmacy, nutritional and fitness status might play even a greater than age itself. However, the available data can be used to give an understanding of the potential modifications in pharmacodynamics and pharmacokinetics of the βAR system with ageing that can help anticipate adverse effects, predict potential interactions with other medications and disease states, guide selection of therapy and titration to target dose.

**Table 1:** **Summary of the experimental evidence focusing on β**_**2**_**AR in ageing**

**Tissue**	**Main experimental finding**	**Potential implications in age-related disorders**
Cardiovascular system	Age-associated decline in βAR sensitivity	Hypertension, Heart failure
Skeletal muscle	β_2_AR agonists rescue age-dependent muscle weakness	Sarcopenia
Airways	Decreased responsiveness to β_2_AR with ageing	Asthma, chronic obstructive pulmonary disease
Brain	Increased β_2_AR density in Alzheimer’s disease	Senile dementia
Immune system	β_2_AR induces a shift towards Th_2_ response	Autoimmune and inflammatory disorders
Pancreatic islets	Impaired β_2_AR-mediated insulin release with age	Diabetes mellitus, glucose intolerance
Liver	β_2_AR density follows a J-shaped curve	Hepatocellular carcinoma
Kidney	Increased β_2_AR density in aged rats	Glomerulosclerosis, hypertension
Bone	β_2_AR stimulates reabsorption of bone tissue	Osteoporosis

### Cardiovascular system

#### Impaired βAR vasorelaxation

Ageing is associated with evident changes in the cardiovascular system [30,31] that presumably reflect perturbations of biochemical adaptive mechanisms [32]. Experimental findings indicate an age-associated decrease in catecholamine-responsiveness in the elderly, documenting a decreased βAR vasorelaxation with ageing [33-35]. Indeed, younger individuals are more responsive than elderly subjects to isoproterenol-induced increases in blood flow in the brachial artery [36]. A large and growing body of scientific evidence has shown that vascular tone is regulated by both the medial (vascular smooth muscle cells, VSMC) and the intimal (endothelial cells, EC) layers, as well as through interlayer interactions [37-40]. Since both VSMC and EC express β_2_AR [41-45], such receptor is expected to play a critical role in age-mediated decline in vasoreactivity [46]. The described decline in adrenergic responsiveness in turn impairs vasodilatation and hastens vasoconstriction, thereby increasing total peripheral resistances. The age-related deterioration in β_2_AR function and subsequent cAMP generation [47-50] is a common factor underlying hypertension, atherosclerosis, vascular insufficiency and orthostatic hypotension, all conditions associated to noteworthy morbidity and mortality [38,51-53]. The increased incidence of atherosclerosis and restenosis with age may also rely on the age-associated deterioration in βAR-mediated cAMP production, since cAMP is considered an inhibitor of VSMC proliferation [54].

Concerning the molecular mechanisms, the age-linked decrease in βAR-mediated relaxation has been proposed to be due to decreased receptor density, less efficient coupling to adenylate cyclase, impaired generation of cyclic AMP, or attenuated activation of downstream components [55]. Variations in cyclooxygenase expression and vasoactive prostanoid levels have been recently implicated [56]. However there is not a single cellular or molecular factor that can fully explain the age-related decline in βAR function and the primary cause of such homeostatic imbalance is yet to be identified. The etiology seems to be associated with an age-related alteration in the ability of βAR to respond to agonists at the cellular level. A fundamental understanding of why βAR-mediated vasodilatation is impaired with age will provide new insights and innovative strategies for the management of the multiple disorders that affect older people [38,44,47,57-59].

An increase in basal levels of circulating catecholamines has been observed with advancing age [60], mirrored by a significant decrease in the number of high affinity βAR [61]. These findings suggest that age-related alterations may be due to βAR desensitization rather than loss of βAR density. βAR affinity for the ligand is dependent upon GPCR phosphorylation, which in turn is in the domain of G protein-coupled receptor kinases (GRKs) and GRK2 in particular [62,63]. Indeed, both GRK2 expression and activity increase in vascular tissue with ageing [64]. Similarly, a generalized impairment of βAR-mediated vasorelaxation has been shown both in animal models of hypertension [58,65] and in human hypertensive patients [62]. Such alteration has been related to an increase in GRK2 abundance and activity [55]: the transgenic overexpression of GRK2 in the vasculature leads to impaired βAR signaling and vasodilatative response, causing a hypertensive phenotype in mice. Such a point of view has been supported in humans by the observation that GRK2 expression correlates with blood pressure as well as impaired βAR-mediated adenylate cyclase activity [66].

#### βAR control of inotropism

An age-associated decrease in βAR sensitivity and density has been shown in the cardiac muscle [31], and has been mainly attributed to down-regulation and impaired coupling of βAR to adenylate cyclase [67]. In particular, a generalized trend toward resting and exertional cardiac output has been reported with advancing age [30,68].

Moreover, a decrease in the catecholamine stimulated adenylate cyclase activity in rat myocardium [69] and in the sensitivity of βARs, measured by isoproterenol-induced changes in pulse rate and blood pressure [70], had been reported. The age-linked decline in cardiac βAR response, which is consistent across species, seems to be primarily due to a down-regulation of β_1_ARs, as reported in aged explanted human hearts [71]. Intriguingly, such feature is similar to what seen in patients with heart failure. Actually, whether ageing causes a selective downregulation of cardiac βAR (β_1_AR *vs* β_2_AR) remains a moot point. Whereas a non-selective decline in both β_1_AR and β_2_AR has been reported in rat senescent cardiac tissue [67], a selective decrease in β_1_AR has been described in ventricular myocytes isolated from aged rats [72]. Many of the modifications that occur in the sympathetic nervous system with ageing (increased circulating catecholamines and hyposensitivity to adrenergic stress, as with exercise, isoproterenol infusion and other agents used to assess cardiovascular reserve) are also common in patients with heart failure [32]. Other potential mechanisms underlying these peculiar aspects are decreased agonist binding of β_1_AR, uncoupling of β_2_AR, involvement of cardiac β_3_AR and abnormal G-protein mediated transduction. Remarkably, unlike heart failure, there is no evidence of upregulation of G_i_ proteins with ageing [67,73]. The compartmentalization of the receptors may also partake in the decreased βAR responsiveness. Indeed, whereas β_1_ARs are widely distributed on the plasma membrane, β_2_ARs are usually located in the transverse tubules, which are invaginations of the plasma membrane containing several proteins that couple membrane depolarization (excitation) to calcium-mediated myofilament shortening (contraction). Thus, the peculiar localization of β_2_AR in cardiac cells leads to the generation of spatially restricted cAMP production, affecting thereby calcium dependent proteins that control the contraction of myofilaments [74,75]. A disrupted localization of β_2_AR has been recently described in chronically failing cardiomyocytes [76], with significant functional sequelae [77]. A similar remodeling of cell surface topography may be involved in ageing, but no specific studies are currently available to confirm such hypothesis. Importantly, conditions presenting a depressed cardiac function elicit activity (fight or flight response [78]) from the sympathetic nervous system to increase cardiac output and to divert blood flow to critical organs. Catecholamines are fundamental actors of this system. The fact that the release of these hormones is strictly controlled by GPCRs relates the adrenal gland and the heart in a ’long-distance affair’ [79,80]. Unfortunately, the relationship between ageing and βARs has not been extensively investigated in chromaffin cells. However, several studies have demonstrated that β_2_AR is definitively involved in the regulation of cathecolamines secretion by the adrenal gland [81-84].

### Skeletal muscle

Ageing is associated with a progressive loss of skeletal muscle mass (sarcopenia) and a subsequent decline in muscle strength [85]. Recent investigations have focused on the underlying mechanisms of age-related effects on skeletal muscle, responsible for the gradual loss of functional independence amongst the elderly. Progressive muscle fibre denervation, a loss of motor units, and potential motor unit remodeling, intrinsic variations in skeletal muscle fibers, including excitation–contraction coupling, have been implicated [86,87].

Skeletal muscle contains all three βAR subtypes, with a ~10 fold greater proportion of the β_2_AR isoform than either β_1_ or β_3_. β_2_ARs are involved in the regulation of contraction, plasma potassium level and glycogenolysis. The available scientific literature concerning the effect of ageing on muscular βARs is controversial and the situation is not so sharp like portrayed above for the cardiac muscle. Whereas some studies imply no age-dependent modification in βAR in skeletal muscle [88,89], other reports suggest an age-related loss in the responsiveness of βAR [35,90]. Indirect evidence for a role of β_2_AR in sarcopenia and ageing comes from works pointing out the capability of specific β_2_AR agonists in reversing age-dependent muscle wasting and weakness. In rats, 4 weeks of fenoterol (a specific β_2_AR agonist) treatment (1.4 mg/kg/day) has been shown to counteract the atrophy and weakness associated with sarcopenia, increasing muscle mass and strength [91]. Of note, fenoterol treatment caused a small increase in fatiguability due to a decrease in oxidative metabolism in both *extensor digitorum longus* and *soleus* muscles. In another recent paper, formoterol treatment has been shown to improve structural and functional regenerative capacity in senescent rats by activation of the mechanistic target of rapamycin (mTOR) [92].

Further investigation is definitively warranted into the mechanisms underlying the relationship between ageing and βARs. In particular, the differences in adrenergic signaling between fast- and slow-twitch skeletal muscles should be assessed. Indeed, the age-linked shift in muscular fiber type proportions (there are more oxidative, type I, fibers in aged tissue) may play a role in such a mechanism. Furthermore, since both α_1_AR and β_2_AR partake in angiogenesis [37,44,93], this issue should be taken in consideration in the studies exploring the potential mechanisms underlying age-associated muscle weakness and fatigue. Lastly, it is unclear whether β_2_AR is responsible for changes in calcium handling and metabolic properties of the muscle. In this sense, the emerging role of mitochondria should be considered [94]. Indeed, decreased mitochondrial content and function have been reported with ageing and might contribute to sarcopenia and chronic disorders. Recent evidence also suggests that mitochondrial biogenesis following aerobic exercise is mediated at least in part through βAR signaling [95].

### Airways

β_2_AR in the airways and lungs are clinically important in a number of disorders, including chronic obstructive pulmonary disease and asthma [96,97]. Studies using different animal models indicate either no change, or a decrease in responsiveness to βAR stimulation with age. In addition, the βAR population has been demonstrated to change with respect to age in different species. A marked increase in βAR number has been shown in late fetal and early post-natal life of rat and rabbit lung [98]. In the rat, this time period coincides with physiological and biochemical changes related to pulmonary maturation. Significant fluctuations in the concentrations of catecholamines, thyroid hormone and corticosteroids have been implicated in the regulation of βAR activity [98]. Several studies have attempted to examine the influence of ageing on responses observed to β_2_AR agonists and other bronchodilators. A decreased βAR agonist affinity and adenylate cyclase activity has been observed in senescent rat lung [99]. Further, age-related changes in the effectiveness of the βAR agonists isoproterenol and fenoterol in both guinea pig and rat isolated tracheal smooth muscle have been reported [100]. An age-associated decrease in βARs has also been described in bovine airways: the quantity of βARs in tracheal epithelium and smooth muscle in cows was 37% and 35% lower, respectively, than in calves [101]. Corroborating these findings, an age-linked reduction in the responsiveness of human subjects to specific β_2_AR agonists has been reported [102]. In particular, response to salbutamol has been investigated in young versus elderly asthmatics, showing a progressive decline in bronchodilation [103].

### Brain

Alterations in the central adrenergic system have been implicated in depression and memory loss, including those suffered by patients with senile dementia. Moreover, adrenergic drugs can improve different aspect of memory loss in ageing animals [104]. An age-related increase in the density of βARs had been reported in the cerebral cortex of both mice [105] and rats [106]. These findings have been also confirmed in aged primates (>20 year-old *rhesus monkeys*), displaying an increase in the density of these receptors in the somatosensory areas and in the primary motor cortex [107]. In humans, examination of postmortem brains has shown that β_2_AR density is elevated in hippocampus and frontal cortex in ageing and in Alzheimer’s disease [108]. Besides, β_2_ARs have been ascertained to play a critical role in the control of behavioral symptoms of Alzheimer’s disease [109]. During the course of the illness, many patients develop indeed aggression, irritability, and agitation [110]. Interestingly, an increase of β_2_AR density has been found in cerebellar subcortical white matter of aggressive demented patients [111]. Furthermore, β_2_ARs in cerebral microvessel fractions from human brain have been found to be significantly increased in Alzheimer’s disease [112]. These studies might help to explain the role of β_2_AR in the pathogenesis of senile dementia and whether treatment with β_2_AR antagonists may provide new therapeutic options for the treatment of Alzheimer’s disease.

Importantly, elderly patients are more susceptible to the psychiatric side-effects of β-blockers than young people [113]. β-blockers may actually cause increased anxiety and agitation [113,114]. Other psychiatric effects common in aged patients include mania, hostility, impulsive behavior and hallucinations [115]. In a recent prospective study conducted in over 5000 subjects with a mean age of 70 years, β-blockers were associated to an increased risk of incident depressive symptoms, especially if non-selective (able to block both β_1_ and β_2_ AR) and lipophilic (able to pass the blood–brain-barrier) [116]. These results suggest a functional role for the age-related modifications in β_2_AR density in the brain. The decreased number of βAR observed in lymphocyte of patients with major depressive disorder [117] and the fact that salbutamol has been found to be as effective as clomipramine in a small trial with depressed inpatients [118], support such a point of view, implying that monoaminergic hyperactivity might be one of the mechanisms underlying the depressive disorder.

### Immunity cells

The immune system has been a focus of intensive gerontological research [51,119,120]. The ability to respond to antigen stimulation declines progressively with age after maturity and cell loss, qualitative cellular alterations (mainly related to the signal-receiving mechanisms) and shift in the proportion of subpopulations have been detected during ageing [121-124]. The earliest report of age-related modifications in human βARs showed a linear decrease in receptor density on lymphocyte membranes taken from subjects between the ages of twenty and eighty years [125]. However, these findings have been successively challenged [126]. Age-associated changes have been described at the receptor and post receptor levels, with parallel modifications in membrane fluidity and capping [24]. A growing body of evidence demonstrates that the complex interaction between the nervous system and the immune system plays a critical role in maintaining homeostasis. Likewise, it is widely known that the nervous system is capable of modulating the immune response via activation of β_2_ARs present on immunocompetent cells.

Over the past three decades, the immunomodulatory properties of β_2_ARs have gained added attention [127]. Alterations in βAR responsiveness have been found in various autoimmune disorders, such as rheumatoid arthritis, multiple sclerosis, myasthenia gravis and type I diabetes mellitus. β_2_ARs have been identified on different immunocompetent cell types and are essential for a number of functions, including cytokine production, natural killer-cell cytotoxicity, and antibody production. Cathecolamine-induced adenylate cyclase activity has been shown to decrease during ageing in human lymphocytes [128]. Moreover, lymphocytes of normal elderly subjects and young asthmatics display dysfunctional βARs [102]. A role for β_2_AR in immune regulation is further supported by the distinctive sequence of its own gene, which comprises a glucocorticoid reactive element (GRE) in the promoter region. Glucocorticoids, commonly used as immunosuppressive drugs to treat autoimmune disease, can indeed upregulate β_2_AR expression [129]. Additionally, infusion of either adrenaline or noradrenaline in human subjects has been shown to modulate the migratory capacity of natural killer cells via a β_2_AR mechanism [130]. Recently, β_2_ARs have been involved, through means of the modulation of cytokine production, in the regulation of defense against extracellular bacteria and the pathogenesis of autoimmune and inflammatory disorders. Activation of β_2_AR results indeed in an inhibition of lymphocyte proliferation and a decrease of IL-2 receptor expression, ultimately leading to a general suppression of the immune reactions [126]. Further, salbutamol has been proved to regulate IL-6 and IL-17 production in murine bone marrow-derived dendritic cells [16,131]. It has been also suggested that the anti-inflammatory nature of βAR stimulation may be the cause of immune response deregulation that is often noted in septic shock [132]. Consequently, β_2_AR activation appears to induce a shift towards a Th_2_-type immune response, inhibiting the production of the Th_1_-type cytokines. The induction of IL-6 observed *in vivo*[133] may be attributed to the β_2_AR stimulation of IL-6 release specifically from adipose tissue, providing thereby a novel mechanism potentially mediating a range of adrenergic effects on energy balance [134,135].

### Pancreatic islets

Impairment of glucose metabolism with age represents a major determinant of type 2 diabetes epidemics within the elderly population [20]. Ageing *per se* is associated with a progressive decrease in basal insulin release, increasing the chance of developing abnormalities in glucose tolerance and overt diabetes mellitus [20,136]. The sympathetic system provides a fine-tuning to the endocrine pancreas activity through αARs and βARs. Furthermore, the reciprocal regulation exerted by insulin and the adrenergic system has been well documented. A role for β_2_AR in the pathogenesis of diabetes has been suggested by the evidence of a decreased number of β_2_AR on granulocytes isolated from type I diabetes patients [137,138]. Studies with β_2_AR agonists imply that β_2_AR might participate in the regulation of insulin secretion [139-141]. In addition, different human polymorphisms in the β_2_AR gene have been associated with obesity and other metabolic disorders [142,143]. More recent evidence demonstrated that β_2_AR knock-out mice display a peculiar phenotype of impaired glucose tolerance, essentially due to reduced insulin secretion from the pancreatic β-cells [20]. In pancreatic islets of Langerhans isolated from these mice PPARγ expression was reduced by 50%, leading to repression of the PPARγ downstream molecules PDX-1 and GLUT2, two key effectors of β-cell function. Importantly, an age-linked decline in β_2_AR levels in mouse pancreatic islets has also been shown [20] and such a feature seems thereby to contribute to the deterioration in glucose tolerance that accompanies ageing.

Glucose metabolism is also modulated by the adipose tissue; βARs, which are involved in the lipolysis and thermogenesis processes, surely partake in such regulation. However, the data concerning the effect of ageing on β_1_AR and β_2_AR density in adipocytes are controversial [103,144-146]. In our opinion, this is likely due to the prominent role of β_3_AR in adipose tissue [10,147-149], together with the fact that such receptor is not identified by the classically used βAR antagonist radioligands.

### Liver

A number of studies demonstrated a biphasic trend in βAR regulation of hepatic glycogenolysis over lifespan: the βAR response declines rapidly during development and re-emerges during senescence [150,151]. Age-related alterations in βAR responsive adenylate cyclase activity follow a J-shaped curve that mirrors the variations in liver glycogenolytic responsiveness. Adrenergic stimulation of glycogenolysis is generally attributed to αAR-mediated processes in young rats and becomes mediated predominantly by βARs during post-maturational ageing [150,152]. Noticeably, in livers from aged rats, β_2_AR density is higher than β_1_AR density [153]. The age-associated increase in βAR gene expression might be due to modifications of the transcription factors involved in regulating the expressions of βAR in the liver. For instance, the early response genes, like c-*myc*, c-*fos* and c-*jun*, are generally thought to partake in regulation of cell proliferation and differentiation, denoting a possible increase in hepatocytes of aged rodents. Consistent with such hypothesis, age-related changes of c*-myc* expression in the mouse follow a J-shaped curve similar to the βAR-sensitive adenylate cyclase activity in the rat liver [154]. Of interest, *in vitro* experiments have shown that βAR density in cultured rat hepatocytes are dependent on cell density [155]. Besides, multiple lines of evidence demonstrate that βAR signaling plays an essential role in the progression and metastasis of cancer and may become a novel target for cancer therapy. A recent report revealed that β_2_AR is upregulated in human hepatocellular carcinoma [156], one of the most common neoplasms and a leading cause of death worldwide [157]. Further investigations are needed to clarify the mechanisms responsible for these alterations in the development and growth of this kind of cancer.

### Kidney

Structural and functional changes in renal function during ageing are among the most dramatic of any tissue [158,159], so that the glomerular filtration rate of healthy octogenarians is only half to two thirds of that measured in young adults [160]. The number of functioning glomeruli declines roughly in accord with the age-linked reduction [161] in renal weight, while the size of the remaining glomeruli increases [162,163]. The importance of genetics in the progressive impairment associated with age has been specifically pointed out in the kidney, with the discovery of the ‘antiageing’ *klotho* gene [164,165]. Mice genetically deficient in *klotho* develop accelerated age-related disorders, including muscle atrophy, osteoporosis, arteriosclerosis and stroke [164]. On the contrary, mice that overexpress *klotho* display a longer lifespan than wild-type rodents [164,166].

Renal βARs are involved in the modulation of both hemodynamics and electrolyte balance [167]. β_2_AR has been shown to regulate the expression of Na/Cl co-transporter expression, thereby participating in the development of salt sensitive hypertension [168,169]. Furthermore, βAR located on the iuxtaglomerular cells mediates renin release [170]. The actual number of β_2_ARs is significantly reduced in membrane preparations of aged rat kidney compared with the young animals [171]. On the other hand, a higher β_1_AR density was found when comparing kidneys from adult to neonatal rats, accompanied by a decrease in Gα_s_ levels [172].

### Bone

Bone remodeling, the mechanism by which vertebrates regulate bone mass, is a process that occurs continuously throughout life to normally maintain bone structure and calcium homeostasis. It comprises two phases, namely the formation by osteoblasts and the resorption by osteoclasts. Such a process is particularly relevant in senile people. Osteoporosis, a condition characterized by low bone mass and increased bone fragility, is indeed one of the most representative age-related disorders in the western world, reducing bone strength and increasing fracture risks [173]. The sympathetic tone has been shown to reduce bone mass by suppressing bone formation and by enhancing bone resorption via activation of the β_2_AR expressed in osteoblasts [174]. In particular, β_2_AR partakes in the osteoanabolic action of parathyroyd hormone (PTH) by controlling expression of PTH-target genes involved in osteoblast activation and bone formation [175]. *In vivo* studies suggest that bone metabolism might be influenced both through indirect activation of βAR signaling via hypothalamic-derived neural pathways and through direct modulation of βAR activity by pharmacological intervention. Indeed, the administration of a specific β_2_AR agonist to rats for 6 weeks led to deleterious effects on trabecular bone microarchitecture, independently from muscle mass [176]. Consistent with these results, reports on β_2_AR knock-out mice [20] have revealed that, as they age, these animals maintain greater trabecular bone microarchitecture, as a result of lower bone resorption and increased bone formation [177]. Another important piece of evidence is the observation that β_1_AR and β_2_AR exert opposite effects on bone: β_1_AR induces a predominant anabolic trigger in response to mechanical stimulation and during growth, whereas β_2_AR mainly regulates bone resorption [177]. Overall, these findings provide new insights into the molecular mechanisms underlying the regulation of bone remodeling by systemic hormones and their local mediators [178].

### Concluding remarks

The present review summarizes the current knowledge about the β_2_AR in ageing. βARs belong to the GPCR family of heptahelical membrane sensors, one of the largest classes of cell-surface receptors, representing essentially the primary target of current pharmaceutical therapies. The function of βAR is modulated by levels of circulating catecholamines, non-catecholamine hormones, drugs, disease and age. Despite many clinical observations demonstrate an age-related decrease in catecholamine responsiveness, the molecular bases of such a phenomenon are still unknown. It is possible and likely that ageing is reflected by a regulation of βAR function at multiple biochemical levels.

Translating these data in the clinical scenario, it is widely accepted that the efficacy of the drugs is different when comparing young and aged populations. In particular, β-blockers have been shown to be more effective in young patients [179]. Moreover, when used as first line treatment of hypertension, β-blockers have similar efficacy to other drugs in younger patients but are less effective than such drugs in older subjects [180,181].

### Future perspectives

Human lifespan has more than doubled in the developed world in the last two centuries. Nevertheless, there are significant gaps in our knowledge of how the process of ageing is initiated and controlled. Undeniably, a better understanding of human longevity will assist in the design of therapeutic strategies to extend the duration of optimal health. In this sense, the mechanisms controlling the selectivity and intensity of the ageing process are likely to be one of the primary goals of biogerontology research in the nearest future. Future investigations addressing the effects of ageing on βAR function and signaling may help to identify new molecular mechanisms to extend and ameliorate age-associated disease, opening new pharmaceutical opportunities for drug discovery in order to achieve a healthy ageing.

## Competing interests

The authors declare that they have no competing interests.

## Authors’ contributions

GS wrote the manuscript. GI had the overall supervision of the review processing. All authors read and approved the final manuscript.
